# Nutritional Risk and Unmet Nutritional Need in Ambulatory Patients Undergoing Systemic Anti‐Cancer Therapies

**DOI:** 10.1111/jhn.70359

**Published:** 2026-10-14

**Authors:** Amy McLuskie, Karen Angel, Matthew Maddocks, Anne Finucane, Marie Fallon, Barry J. A. Laird, Michael S. Yule

**Affiliations:** ^1^ Institute of Genetics and Cancer University of Edinburgh Edinburgh UK; ^2^ Western General Hospital Edinburgh UK; ^3^ Cicely Saunders Institute of Palliative Care Policy and Rehabilitation, King's College London London UK; ^4^ Clinical Psychology, Health in Social Science University of Edinburgh Edinburgh UK; ^5^ Marie Curie Hospice Edinburgh UK; ^6^ Institute of Clinical Medicine, Medical Faculty University of Oslo Oslo Norway; ^7^ Department of Oncology, European Palliative Care Research Centre (PRC) Radium Hospital, Oslo University Hospital Oslo Norway; ^8^ St Columba's Hospice Care Edinburgh UK

**Keywords:** cancer, malnutrition, nutritional screening, prevalence, survival, toxicity

## Abstract

**Background and Aims:**

Malnutrition is highly prevalent among cancer patients receiving systemic anti‐cancer therapy (SACT) and is associated with poor treatment tolerance, reduced quality of life, and increased mortality. Despite recommendations, nutritional screening in the outpatient setting is inconsistently implemented and the prognostic significance of nutritional risk identified using a validated screening tool is still unclear. This study aimed to determine the prevalence of nutritional risk among patients receiving SACT, whether their nutritional needs were being met with dietetic input, and to examine associations with clinical outcomes, including treatment toxicity, dose reduction, hospital admission, and overall survival.

**Methods:**

In this prospective observational study, adult outpatients receiving SACT were invited to complete the Patient‐Generated Subjective Global Assessment Short Form (PG‐SGA SF), a nutritional screening tool. Nutritional risk using PG‐SGA SF scores was assessed in relation to treatment toxicity, dose reduction, and hospital admission using multivariable regression models and survival was evaluated using Cox proportional hazards models, both adjusted for age, sex, metastatic disease, and performance status.

**Results:**

Of 169 participants, 24.9% were at nutritional risk, PG‐SGA SF score 4–8, and 10.7% at high risk ≥ 9, yet only 5.3% were in receipt of dietetic care. Higher PG‐SGA SF scores were independently associated with increased treatment toxicity (OR: 1.2, 95% CI: 1.1–1.3, *p* = 0.003) and SACT dose reductions (OR: 1.2, 95% CI: 1.0–1.3, *p* = 0.040). Nutritional risk was not significantly associated with 30‐day hospital admissions but was independently associated with overall survival (HR: 1.1, 95% CI: 1.0–1.2, *p* = 0.004).

**Conclusions:**

Nutritional risk identified using the PG‐SGA SF independently predicted treatment toxicity, dose reduction, and survival in those receiving SACT. Despite over one‐third of those meeting the criteria for dietetic intervention, only 5.3% were receiving dietetic care, indicating a substantial gap between dietetic need and provision. These results support implementation of routine screening, referral pathways, and a dedicated dietetic service integrated into oncology outpatient services.

## Key points

- It was identified that there was an unmet nutritional need among those attending for SACT and the requirement for a dedicated dietetic service.
- Nutritional risk was prevalent, yet few were in receipt of dietetic care.
- Routine screening should be implemented for those attending oncology outpatient services, using a validated screening tool such as the PG‐SGA SF.

## 1 Introduction

Malnutrition is prevalent in cancer populations and contributes to reduced treatment tolerance, impaired quality of life (QoL), and reduced survival [1, 2]. Malnutrition occurs in 40%–60% [2, 3] of people with cancer, with this increasing to 80% in those with advanced disease [4]. Cancer and its treatment can significantly impact the nutritional status of patients, leading to increased risk of complications [5]. The consequences of malnutrition in this population can be catastrophic, with an estimated 10%–20% of patients with cancer dying due to a consequence of malnutrition rather than due to the tumour itself [6]. Optimal nutritional care is becoming increasingly recognised as a vital component of modern cancer care.

The European Society for Clinical Nutrition and Metabolism (ESPEN) promotes the screening and assessment of all patients for nutritional disturbances, providing recommendations tailored to the specific cancer type, treatment, and stage [7]. Therefore, nutritional screening and support should be initiated at the point of diagnosis [1, 8], with screening continued routinely, as indicated [7, 9]. Despite guideline recommendations, nutritional screening is inconsistently implemented in outpatient oncology settings [1, 10], where malnutrition is common [1]. Previous studies have indicated that access to support is limited, and in the United Kingdom, only 36% of people with cancer who require early nutritional intervention are referred to a dietitian [11]. This has also been identified in other European countries, with only one in three cancer patients at risk of malnutrition receiving nutritional support [12]. In a French study, 41% of those identified at nutritional risk received nutritional counselling [10, 13]. Despite screening recommendations, it remains unclear how nutritional risk is identified during routine outpatient consultations and how nutritional risk relates to clinically important outcomes such as treatment tolerance and survival.

Timely nutritional screening and early identification of nutritional risk enable referral to dietetic services and are associated with improved clinical outcomes and QoL [14, 15, 16]. Nutritional screening tools should be validated, standardised and easy to use in clinical practice [10]. The Patient‐Generated Subjective Global Assessment (PG‐SGA) is a validated oncology screening tool that can be used in outpatient settings [10, 17]. The short form, the abridged PG‐SGA, is completed by the patient and covers four domains: weight, food intake, symptoms, and physical function. It scores malnutrition risk accordingly and indicates whether nutritional intervention is required, depending on risk and symptoms. Numerical scores range from 0 to 36, and a score ≥ 9 indicates high risk. In a subsequent evaluation of screening tools within the same outpatient cohort, it was shown that the PG‐SGA SF identifies broader dimensions of nutritional risk than anthropometric screening tools alone, supporting its role as a nutritional screening and triage tool [18]. Those identified as at nutritional risk, as per the recommendations, should be referred to a dietitian for a nutritional assessment and an individualised nutritional care plan [1, 10]. Despite the recommendation of routine nutritional screening, few studies have examined whether nutritional risk identified during outpatient appointments independently predicts clinically important outcomes whilst simultaneously quantifying the gap between nutritional risk and dietetic service provision. Addressing this is important for informing the implementation of routine nutritional screening within oncology services. This study aimed to quantify the prevalence of nutritional risk among those attending for SACT, assess whether nutritional needs are being met through access to specialist dietitians, and evaluate the need for a dedicated dietetic service.

Secondary aims were to evaluate the clinical relevance of nutritional screening and examine associations between nutritional risk and the following clinical outcomes: toxicity, dose reduction, 30‐day hospital admission, and survival.

## 2 Materials and Methods

### 2.1 Study Design and Setting

This study was a prospective observational cohort study conducted at the day therapy unit of the Cancer Centre, a specialist regional facility. It serves a catchment of 1.4 million people across the South East of Scotland and is one of three regional cancer networks serving Scotland [19].

### 2.2 Participants

All adult patients (≥ 18 years) with a solid tumour, receiving SACT between 6 and 31 January 2025, were invited to complete the PG‐SGA short form (SF) and additional questions on dietetic input and oral nutritional supplement (ONS) use, as a convenience sample. Potential participants were offered the questionnaire by the receptionist whilst they were waiting to attend the ward for treatment. Questionnaires were then returned to the receptionist and collected by the oncology dietitian. Patients attending the unit for the first time to commence their treatment were not invited to complete the questionnaire whilst waiting in the waiting area. This was due to the observational nature of the study, with no referrals made based on the outcome of nutritional screening and no staff involvement to assist in completing the questionnaire. This was done to prevent any unnecessary additional distress whilst they were waiting to start their first dose of SACT. Otherwise, all eligible patients attending the unit during the study period were considered for inclusion to minimise selection bias. A power calculation was not conducted as the study aimed to include all eligible patients attending the unit within the predefined study period. SACT was defined as: neoadjuvant, adjuvant, or palliative treatment, including chemotherapy and/or immunotherapy and/or targeted therapy and/or hormonal therapy. This selection aimed to include a broad spectrum of patients, with both metastatic and non‐metastatic disease. Those who were not receiving SACT or were unable to fully complete the questionnaire were excluded from the analysis.

### 2.3 Nutritional Risk Screening Method

The PG‐SGA SF is a validated screening tool specifically designed for oncology populations [20]. The PG‐SGA SF evaluates recent weight change, dietary intake, nutrition impact symptoms, and functional capacity, allowing categorisation of patients according to nutritional risk. The pre‐determined scoring system was utilised [20]: a score of 0–1, no intervention is required; 2–3, intervention by a dietitian, nurse, or clinician, with pharmacological intervention dependent on symptoms; 4–8, intervention by a dietitian and another clinician, depending on symptoms; and ≥ 9, critical need for improved symptom management and/or nutrient intervention [20]. Additional questions in the questionnaire examined whether participants at risk of or experiencing malnutrition had been referred to or reviewed by a dietitian, and if they were using ONS.

### 2.4 Data Collection

Following the return of a completed questionnaire, clinical and demographic data were extracted from electronic patient records. Data extracted included age, sex, primary tumour site, and metastatic status. Data on treatment type, toxicity, dose reduction, 30‐day hospital admission, and neutrophil‐to‐lymphocyte ratios (NLR) were provided by the Cancer Information Services. An NLR ≥ 3.5 was considered higher, in line with previous research [21]. When multiple NLR results were present, the value closest to 15 January 2025 was extracted. The maximum toxicity grade across all toxicities (constipation, diarrhoea, fatigue, nausea, neurotoxicity, oesophagitis, vomiting, and oral mucositis) was calculated and categorised as an ordinal variable (0, 1, ≥ 2). Toxicity was graded according to the National Cancer Institute Common Terminology Criteria for Adverse Events (NCI CTCAE) [22, 23], and for performance status (PS), the Eastern Cooperative Oncology Group (ECOG) scale was employed [24, 25]. Participants were followed up for 1 year. This observational study used prospectively collected data obtained during dietetic service evaluation undertaken within routine clinical practice. The project was reviewed and approved through local NHS governance processes in accordance with institutional quality improvement policies. Formal Research Ethics Committee review was not required as the project involved service evaluation and did not alter standard patient care. Participation was voluntary, and completion of the questionnaire was taken as implied consent. All the information was anonymised prior to analysis. Data were stored securely in accordance with local data protection and confidentiality procedures.

### 2.5 Statistical Analysis

Continuous variables are presented as mean and standard deviation (SD) or median and interquartile range (IQR), based on visual inspection for normality. Categorical variables are presented as frequencies and percentages [*n* (%)]. The PG‐SGA SF score was analysed as a continuous variable for clinical outcomes and as a dichotomous categorical variable, in line with the PG‐SGA SF cut‐off for nutritional risk (< 4 or ≥ 4) for survival analysis. Body Mass Index (BMI) was calculated (weight [kg]/squared height [metres^2^]) and categorised as per PG‐SGA SF criteria [20]. Missing data were handled using complete case analysis. Associations between nutritional risk and outcomes were analysed using ordinal regression (toxicity), binary logistic regression (dose reduction and hospital admission), and survival was assessed using Kaplan–Meier methodology, log‐rank tests and Cox proportional hazard models. Follow‐up time was calculated from 6th January to the date of death (event) or the date of last contact (censored). Models were adjusted for clinically plausible confounders (age, sex, metastatic disease, and ECOG PS). All statistical analyses were performed using IBM SPSS Statistics Version 29.0.1.0. A *p*‐value of ≤ 0.05 was considered statistically significant. Survival visualisations were conducted using R (v4.4.1) and RStudio (v2026.1.0.392), using the *tidyverse, survival*, and *survminer* packages.

## 3 Results

### 3.1 Participants

A total of 189 questionnaires were returned by participants. One questionnaire was excluded due to insufficient data for analysis, and a further 19 were excluded as participants were not currently in receipt of SACT. This resulted in 169 participants for analysis (Figure 1). Of the included participants, sufficient data to calculate a PG‐SGA SF score were available for 131 (77.5%) participants. There were 22 (13.0%) participants with metastatic disease and 16 (9.5%) with non‐metastatic disease; insufficient data were available to calculate a PG‐SGA SF score. For participants with missing height or weight, data were obtained from clinical records (*n* = 2), allowing PG‐SGA SF scores to be calculated for 133 participants (78.7%). Regression models were conducted using complete cases (*n* = 125–133, depending on the outcome).

**Figure 1:**
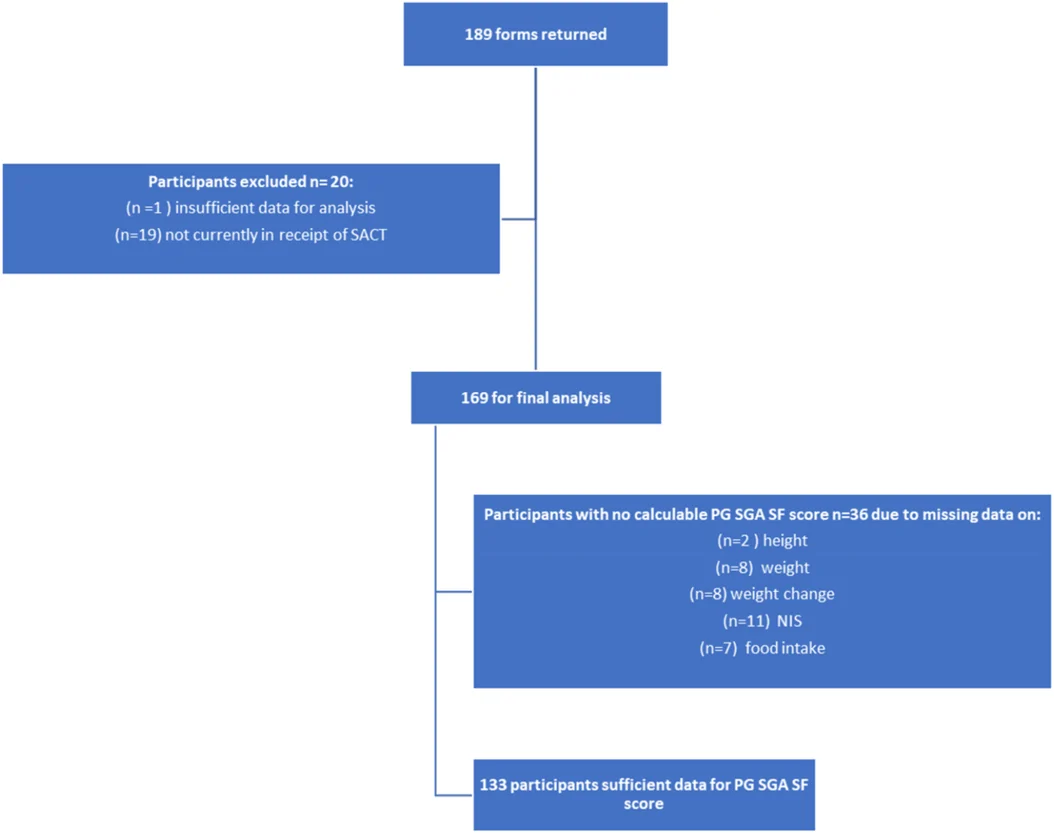
Study flowchart. PG‐SGA SF, Patient‐Generated Subjective Global Assessment Short Form; NIS, Nutrition Impact Symptoms; SACT, Systemic Anti‐Cancer Therapy.

The cohort comprised 130 (76.9%) females and 39 (23.1%) males with a median age of 62 years (IQR: 53.0–69.0). The most prevalent tumour types were breast (*n* = 69, 40.8%), gynaecological (*n* = 24, 14.2%), gastrointestinal (*n* = 32, 18.9%), and lung (*n* = 15, 8.9%). Metastatic disease was present in 58.6% of participants. Median body mass index (BMI) was 25.4 kg/m^2^ (IQR: 23.2–29.5 kg/m^2^). Full demographic and clinical characteristics are presented in Tables 1 and 2.

**Table 1:** Demographic and clinical characteristics of study cohort.

Variable	Level	Number (%)
Age	Median (IQR)	65.5 (53.0–69.0)
Gender	Female	130 (76.9)
	Male	39 (23.1)
Performance status	0	96 (56.8)
	≥ 1	67 (39.6)
	Missing	6 (3.6)
PG‐SGA score	0–1	52 (30.7)
	2–3	21 (12.4)
	4–8	42 (24.9)
	≥ 9	18 (10.7)
	Missing	36 (21.3)
BMI	< 18.5	4 (2.4)
	18.5–20	8 (4.7)
	20–25	59 (34.9)
	25–30	52 (30.7)
	> 30	36 (21.3)
	Missing	10 (5.9)
1‐month weight loss	< 5%	36 (21.3)
	5%–10%	6 (3.6)
	> 10%	1 (0.6)
	Weight gain	100 (59.2)
	Missing	26 (15.4)
6‐month weight loss	< 5%	37 (21.9)
	5%–10%	26 (15.4)
	> 10%	9 (5.3)
	Weight gain	65 (38.5)
	Missing	32 (18.9)
Tumour group	Brain	6 (3.5)
	Breast	69 (40.8)
	Gastrointestinal	32 (18.9)
	Gynaecological	24 (14.2)
	Head and neck	6 (3.6)
	Lung	15 (8.9)
	Urological	9 (5.3)
	Other	8 (4.7)
Metastatic disease	Yes	99 (58.6)
	No	70 (41.4)
Albumin (g/L)	Mean (SD)	34.56 (±3.02)
	Missing	37
CRP (mg/L)	Median (IQR)	7.5 (3.0–53.0)
	Missing	145
NLR	Low	118 (69.8)
	High (≥ 3.5)	38 (22.5)
	Missing	13 (7.7)
Hospital admission	Yes	40 (23.7)
	No	129 (76.3)
Treatment toxicity	0	31 (19.1)
	1	108 (63.9)
	≥ 2	23 (13.6)
	Missing	7 (4.1)
1‐year survival	Yes	134 (79.3)
	No	35 (20.7)
Dose reduction	Yes	20 (11.8)
	No	149 (88.2)
Treatment type	Chemotherapy	81 (47.9)
	Targeted therapy	28 (16.6)
	Immunotherapy	26 (15.3)
	Combination	30 (17.8)
	Hormonal	4 (2.4)

Abbreviations: BMI, body mass index; CRP, C‐reactive protein; NLR, neutrophil:lymphocyte ratio; PG‐SGA, Patient‐Generated Subjective Global Assessment.

**Table 2:** Frequency of toxicities and grade.

Toxicity type	Toxicity grade	Number (%)
Constipation	0	125 (74)
	1	29 (17.2)
	2	7 (4.1)
	3	1 (0.6)
	Missing	7 (4.1)
Diarrhoea	0	144 (85.2)
	1	14 (8.3)
	2	4 (2.4)
	Missing	7 (4.1)
Nausea	0	118 (69.8)
	1	41 (24.3)
	2	3 (1.8)
	Missing	7 (4.1)
Neurotoxicity	0	112 (66.3)
	1	44 (26.0)
	2	6 (3.6)
	Missing	7 (4.1)
Oesophagitis	0	132 (78.1)
	1	30 (17.8)
	Missing	7 (4.1)
Vomiting	0	154 (91.1)
	1	7 (4.1)
	2	1 (0.6)
	Missing	7 (4.1)
Oral mucositis	0	138 (81.7)
	1	22 (13)
	2	2 (1.2)
	Missing	7 (4.1)

### 3.2 Prevalence of Nutritional Risk and Unmet Nutritional Need

Mean PG‐SGA SF score was 4.1 (SD: ±4.0). Overall, 42 (24.9%) participants had a PG‐SGA SF score of 4–8, indicating nutritional risk warranting intervention by a dietitian, alongside a nurse or physician, depending on symptoms. There were 18 (10.7%) at high risk (≥ 9) requiring nutrient intervention and/or symptom self‐management (Table 1). Despite over a third of patients meeting the criteria for dietetic intervention, only 5.3% were in receipt of dietetic care at the time of the evaluation (Table 3). Oral nutritional supplement (ONS) use was reported by seven (4.1%) participants. Of these, three were under dietetic care and had ONS prescribed, while four participants were not under dietetic care and had purchased supplements independently. The most prevalent NIS were anorexia (*n* = 41), taste changes (*n* = 31), and fatigue (*n* = 27).

**Table 3:** Characteristics of those in receipt of dietetic input.

Diagnosis	BMI	Weight changes 1 month	Weight changes 6 months	Nutrition impact symptoms	Oral nutritional supplements	PG‐SGA score
Metastatic ovarian	> 20	< 5%	5%–10%	No appetite	Prescribed	8
Metastatic breast	> 25	Stable	5%	—	Prescribed	—
Metastatic oesophageal	—	—	—	Eating less, nausea, and taste changes	No	—
Head and neck*	< 18.5	< 5%	Gain	Swallow	No	—
Ovarian	> 20	Stable	Stable	Nil	No	2
Head and neck	18.5–20	Gain	—	Other	No	4
Pancreatic	> 25	Gain	Gain	Satiety	Prescribed	—
Oesophageal	—	Stable	5%–10%	Taste changes, swallow, satiety, and fatigue	No	7
Head and neck	> 20	Gain	< 5%	No appetite and nausea	No	8

* Tube fed.

### 3.3 Relationship Between Nutritional Status and Patient Outcomes

Higher PG‐SGA SF scores (*n* = 126) were significantly associated with increased toxicity (*p* = 0.002). In the adjusted ordinal regression, PG‐SGA SF (*n* = 125) remained an independent predictor of toxicity (OR: 1.2, 95% CI: 1.1–1.3, *p* = 0.003) (Table 4).

**Table 4:** Ordinal regression showing the relationship between toxicity and nutritional risk.

Co‐variable	Regression coefficient	Standard error	*p*	OR	95% CI	Single score test (*p*)
Thresholds						
Intercept1 (Grade 0)	0.098	1.223	0.753	—	—	—
Intercept2 (Grade 1)	3.719	1.279	0.009	—	—	—
Predictors						
Age	0.009	0.017	0.580	1.009	0.975–1.041	0.463
PG‐SGA score	0.158	0.053	0.003	1.171	1.055–1.300	0.002
ECOG PS (0 vs. ≥ 1)	−0.597	0.407	0.163	0.550	0.236–1.273	0.022
Sex (Male vs. Female)	0.814	0.470	0.087	2.14	0.888–5.973	0.355
Metastases (No vs. Yes)	0.274	0.408	0.506	1.256	0.566–2.792	0.510

*Note:* For categorical variables, the reference category is shown in brackets. PG‐SGA, Patient‐Generated Subjective Global Assessment; ECOG‐PS, European Co‐operative Oncology Group Performance Status.

Higher PG‐SGA SF score and increasing age were associated with dose reduction. In the multivariate analysis, PG‐SGA SF score (*n* = 127, dose reduction *n* = 15) independently predicted dose reduction (OR 1.2, 95% CI 1.0–1.3, *p* = 0.040). There were no significant associations between PG‐SGA SF score and gender, performance status (ECOG), or metastatic disease (Table 5).

**Table 5:** Binary logistic regression showing the relationship between dose reduction and nutritional risk.

Variable	Univariate			Multivariate		
	OR	95% CI	*p*	OR	95% CI	*p*
Age	1.05	1.01–1.100	0.029	1.09	1.02–1.16	0.009
Sex (Male vs. Female)	0.89	0.30–2.62	0.828	0.69	0.18–2.69	0.595
Metastases (No vs. Yes)	0.85	0.33–2.17	0.729	0.36	0.11–1.23	0.102
PG‐SGA score	1.11	0.98–1.25	0.102	1.16	1.01–1.34	0.040
ECOG PS (0 vs. ≥ 1)	1.62	0.79–3.32	0.190	0.81	0.24–2.81	0.745

*Note:* For categorical variables, the reference category is shown in brackets. PG‐SGA, Patient‐Generated Subjective Global Assessment; ECOG‐PS, European Co‐operative Oncology Group Performance Status.

PG‐SGA SF score was not significantly associated with 30‐day admission to hospital during the observed period (February–March 2025) (*n* = 127, admissions *n* = 28) (OR: 1.1, 95% CI: 0.1–1.2, *p* = 0.16), after adjusting for age, gender, metastatic disease, and ECOG. There was a significant association between hospital admission and gender (OR: 0.3, 95% CI: 0.1–0.7, *p* = 0.013) (Table 6).

**Table 6:** Binary logistic regression showing the relationship between 30‐day hospital admission and nutritional risk.

Variable	Univariate			Multivariate		
	OR	95% CI	*p*	OR	95% CI	*p*
Age	1.10	0.98–1.04	0.396	1.02	0.98–1.06	0.429
Sex (Male vs. Female)	0.52	0.238–1.15	0.109	0.28	0.103–0.772	0.013
Metastases (No vs. yes)	0.83	0.40–1.69	0.599	0.74	0.28–1.95	0.558
PG‐SGA score	1.07	0.97–1.185	0.174	1.09	0.97–1.21	0.160
ECOG PS (0 vs. ≥ 1)	1.67	0.79–3.12	0.190	1.92	0.74–5.00	0.179

*Note:* For categorical variables, the reference category is shown in brackets. PG‐SGA, Patient‐Generated Subjective Global Assessment; ECOG‐PS, European Co‐operative Oncology Group Performance Status.

The 1‐year mortality rate across the sample was 22.1% (95% CI: 15.3–28.3). A total of 127 patients were included in the multivariate Cox regression model. Higher PG‐SGA SF score was independently associated with an increased hazard of death (HR: 1.1, 95% CI: 1.0–1.2, *p* = 0.004). A worse ECOG PS was also independently associated with mortality (HR: 3.1, 95% CI: 1.4–6.9, *p* = 0.007), as was metastatic disease (HR: 8.8, 95% CI: 2.4–31.9, *p* < 0.001). Female patients had significantly lower mortality than male patients (HR: 0.3, 95% CI: 0.1–0.7, *p* = 0.003) in this cohort. There was no significant association with age (HR: 0.9, 95% CI: 0.9–1.0, *p* = 0.209) (Table 7). Unlike in the continuous PG‐SGA SF score the dichotomised score (< 4 vs. ≥ 4) there was no significant difference in survival probability between those categorised as low‐risk and high‐risk (Figure 2).

**Table 7:** Multivariable Cox proportional hazards model.

Variable	Univariate			Multivariate		
	HR	95% CI	*p*	HR	95% CI	*p*
Age	1.010	0.982–1.039	0.490	0.976	0.940–1.014	0.209
Sex (Male vs. Female)	0.383	0.194–0.753	0.005	0.307	0.139–0.677	0.003
Metastases (No vs. Yes)	5.994	2.116–16.983	< 0.001	8.783	2.416–31.923	< 0.001
PG‐SGA score	1.083	1.000–1.174	0.051	1.140	1.044–1.245	0.004
ECOG PS (0 vs. ≥ 1)	2.705	1.363–5.372	0.004	3.069	1.362–6.916	0.007

*Note:* For categorical variables, the reference category is shown in brackets. PG‐SGA, Patient‐Generated Subjective Global Assessment; ECOG‐PS, European Co‐operative Oncology Group Performance Status.

**Figure 2:**
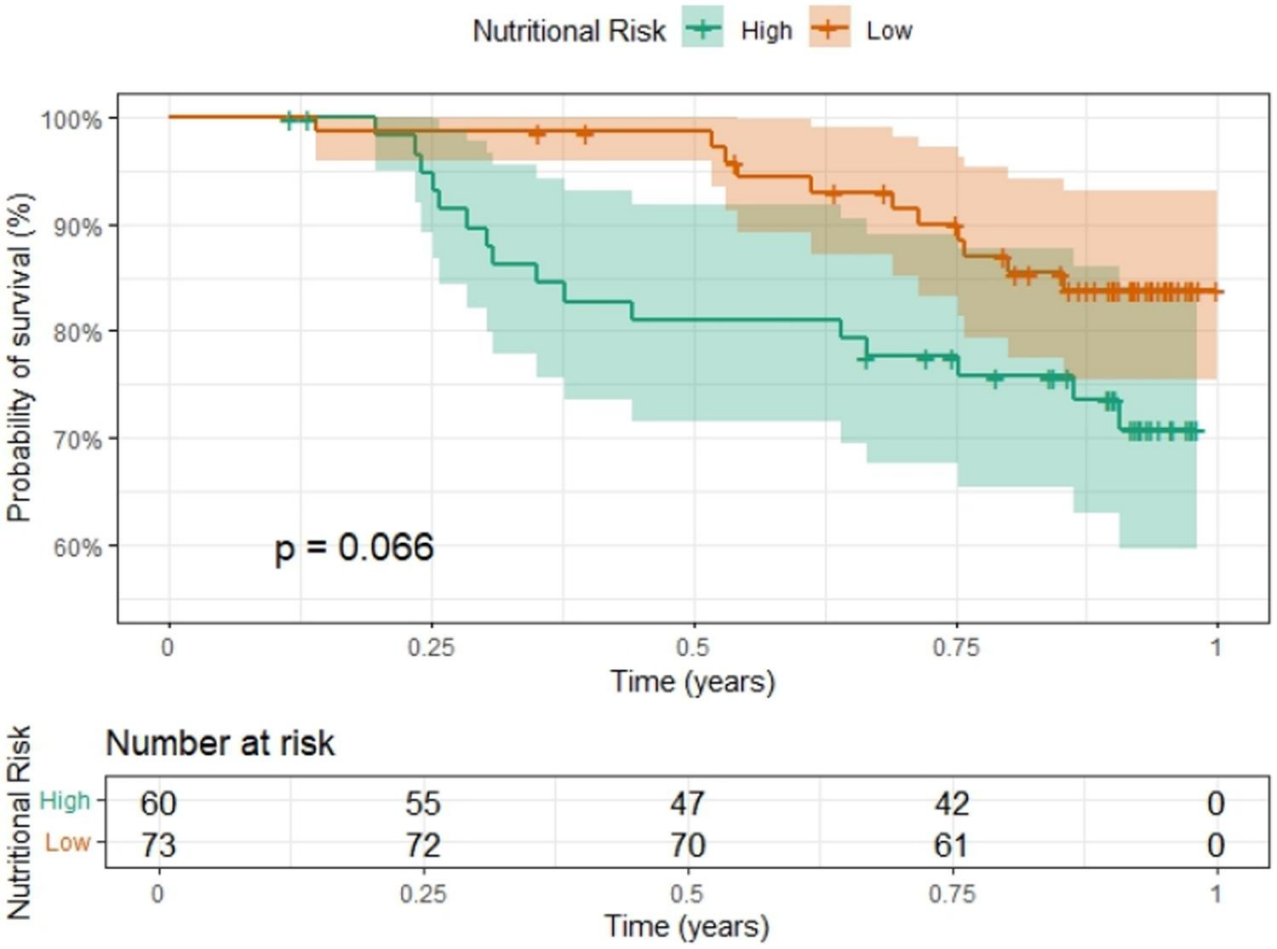
Kaplan–Meier curve showing the difference in survival between high nutritional risk (PG‐SGA ≥ 4) and low nutritional risk (PG‐SGA < 4).

## 4 Discussion

This study demonstrated four main findings. Firstly, nutritional risk was prevalent among those receiving SACT, with more than a third meeting the criteria for dietetic referral and/or intervention. Secondly, despite this high prevalence, very few were receiving dietetic input, evidencing a gap in dietetic need and provision. Thirdly, nutritional risk independently predicted clinical outcomes and overall survival in the adjusted regression models. Finally, the results from this study show the importance of routine screening with a validated screening tool to identify nutritional risk and for supportive, integrated oncology care that includes dietitians.

There were 24.9% of those with a calculable PG‐SGA SF score who were identified as at nutritional risk [4, 5, 6, 7, 8], with a further 10.7% identified as at high risk (≥ 9), yet only 5.3% of the sample (*n* = 169) received dietetic input. The gap between need and service provision is consistent with previous research [10] demonstrating under‐referral to dietetic services in oncology populations. The small proportion of participants receiving dietetic input in this cohort indicates nutritional concerns remain under‐recognised and under‐treated in routine clinical practice [26, 27], especially in outpatient settings where resource limitations may prevent the implementation of routine nutritional screening [1, 28]. This study expands previous work emphasising an unmet clinical need and providing evidence for integrating a dedicated dietetic service into the multidisciplinary team for those attending for SACT [29]. The complexity of patients' needs with a cancer diagnosis requires a multimodal, multidisciplinary team approach which includes oncologists, palliative medicine, dietitians, nurses, allied healthcare professionals, and psychologists [30]. Incorporating the key elements of nutritional counselling, pharmacology, exercise, and psychological support can help identify and treat patients at risk of malnutrition whilst providing more accessible and efficient care [30]. The present study indicates that nutritional risk identified in routine practice has implications for clinical outcomes, along with survival and identifies an unmet nutritional need. Despite this study being conducted in a single centre, previous studies and international guidelines have indicated unmet nutritional needs and the need for implementing routine nutritional screening [1, 6, 10, 11, 12]. It is important to consider why patients are not being referred, whether this is due to a lack of routine screening, resources, or awareness of the implications of nutritional risk in oncology patients amongst healthcare professionals.

In a related diagnostic performance study conducted in the same cohort, the PG‐SGA SF and Malnutrition Universal Screening Tool (MUST) were compared against the Global Leadership Initiative on Malnutrition (GLIM) criteria for malnutrition. It was demonstrated that MUST had greater specificity; however, the PG‐SGA SF identified a wider cohort with NIS and functional impairment that may be missed with screening tools that solely use anthropometric measures. Together, these results strengthen the requirement for implementing routine nutritional screening that incorporates patient‐reported outcomes, identifying those in need of dietetic input and as a triage for supportive and integrated care [18].

Higher PG‐SGA SF scores were independently associated with increased treatment‐related toxicity. Malnutrition and associated reduced muscle mass may impair tolerance to SACT [31, 32]. Previous studies have demonstrated associations between poor nutritional status, low skeletal muscle mass (SMM), and increased chemotherapy toxicity. The present results contribute to this evidence by indicating that the PG‐SGA SF can identify those at increased risk of treatment complications in the outpatient setting. Body surface area (BSA) is used for chemotherapy dosing, but those with similar BSAs can differ greatly in lean body mass (LBM) [31]. Low SMM is associated with chemotherapy toxicity, reduced time to tumour progression, poor physical function, and increased mortality [12, 31]. Muscle loss and sarcopenia can be present even in those categorised as overweight or obese, making detection using routine measurements challenging [12]. Computed tomography (CT) is the gold standard for measuring muscle mass, but it is limited to research settings; in such cases, screening tools such as the PG‐SGA SF can provide subjective estimates of muscle stores [33, 34].

Higher nutritional risk was independently associated with dose reduction. These data support further evaluation regarding earlier dietetic intervention and the effects on treatment tolerance. Dosing of chemotherapy based on LBM is an emerging area of research. A recent study found that LBM‐based oxaliplatin dosing in colon cancer reduced peripheral neurotoxicity and improved QoL without compromising survival [32]. A meta‐analysis further confirmed an association between low SMM and severe treatment‐related toxicity, with higher rates of physician‐requested dose reduction or therapy termination in those with low SMM [31]. In a study of patients with upper gastrointestinal diseases, a PG‐SGA‐SF score of ≥ 9 at diagnosis was associated with a fourfold increased risk of losing 5.0% muscle mass, with each 1.0% of weight loss corresponding to a 1.2% loss of muscle mass, a potentially modifiable factor [35], emphasising the importance of nutritional screening to identify those at high nutritional risk and the implications of this on treatment‐related outcomes.

In contrast, nutritional risk was not significantly associated with 30‐day hospital admission. This may be due to the limited number of hospital admissions. The direction of association observed in this study is consistent with previous literature, linking malnutrition to greater healthcare utilisation [1, 29]. This can be attributed to those with low muscle mass, a symptom of malnutrition occurring frequently. This can have multiple causes, such as reduced oral intake, decreased physical activity, and/or cancer treatments. Those with low muscle mass can experience decreased physical function and QoL, leading to longer hospital stays, unplanned admissions, and re‐admissions [29, 36].

Higher PG‐SGA SF scores were associated with decreased overall survival. These findings show that nutritional risk provides independent prognostic information beyond established oncological indicators, such as performance status. These results are consistent with previous studies demonstrating that malnutrition and muscle depletion predict reduced survival [12, 28, 37].

There were a number of participants classified as overweight (30.7%) or obese (21.3%), similar to other studies with mixed cohorts [28]. Weight gain in our participants at 1 and 6 months was reported in 59.2% and 38.5%, respectively. This was likely due to the high number of breast cancer participants, who commonly receive endocrine therapies and steroids [38]. Weight gain can also be a concern among those treated with SACT and can remain unaddressed [38]. Previous studies suggest that many patients are classified as overweight or obese, even when metastatic disease is present [39]. Those who are not visibly underweight despite significant weight loss may not be identified as at nutritional risk, depending on how nutritional risk is assessed. Sarcopenic obesity is an indicator of poor survival, emphasising the importance of nutritional screening [28, 37]. The PG‐SGA SF may help identify nutritional risk across the BMI categories. One study found that sarcopenia and myosteatosis, identified by CT, were present across a range of nutritional risk scores, showing the additional prognostic value of CT‐derived SMM analysis alongside PG‐SGA [37]. These findings reinforce the importance of nutritional screening that goes beyond body weight alone.

The most prevalent NISs in our cohort were anorexia, taste changes and fatigue. Previous studies have shown anorexia to be associated with poor QoL and reduced survival in patients with advanced cancer [28, 40]. Tong et al. [41], assessed the prevalence of NIS and QoL in oncology patients, where a similar number of cancer patients were identified with a PG‐SGA score ≥ 9, (12.3%) after 1 month of commencing treatment, increasing at 6 months (14.2%), with only half of this cohort having dietetic input [41].

Nutritional screening in the outpatient setting can be inconsistent or absent [1, 42]. The benefits of using a validated patient‐reported screening tool such as the PG‐SGA SF include capturing patient‐reported symptoms and alleviating the resource limitations faced by oncology staff conducting nutritional screening in busy oncology units [43]. The present study shows the feasibility of a patient‐generated screening tool, as indicated by a high completion rate (78.7%) without staff support or prior training. This would decrease the workload of clinical staff and promote a patient‐centred approach [43]. There is no agreement on which screening tool to use to identify nutritional risk. The PG‐SGA SF can be used as a screening, initial assessment, triage, and monitoring tool [30]. Using an integrated team approach to implement a screening tool, such as the PG‐SGA SF, quickly identifies those at risk of malnutrition, facilitates targeted treatment by the most appropriate clinician, and aims to prevent malnutrition and its adverse effects [39]. Previous studies have shown that the PG‐SGA SF requires little completion time for patients and that only 26% of patients required assistance [39, 44]. Fu et al. (2022) [45] assessed the validity of a modified PG‐SGA tool. While 69.0% of healthcare professionals expressed concern that it may be too difficult for patients or their families to complete independently, 99.0% agreed that patients or their families should complete it independently. In the present study, no staff training or support was provided, yet completion rates remained high. Nonetheless, staff input would be required to minimise completion bias and ensure accuracy, a potential barrier in outpatient settings [1].

### 4.1 Strengths and Limitations

Of those who completed the survey, 89.4% were included in the main analysis and over half (58.6%, *n* = 99) had metastatic or incurable disease. The present study had several notable strengths. It represents real‐world data from an outpatient oncology setting with a heterogeneous cohort receiving SACT, across several tumour groups and treatment intents. The PG‐SGA SF score was used as a continuous variable to support the detection of dose‐response relationships and to demonstrate that incremental deterioration in nutritional status was associated with progressively worse outcomes. Analysis adjusted for key confounders, including age, ECOG PS, metastatic disease, and sex. The inclusion of multiple clinically relevant end‐points (treatment‐related toxicity, dose reduction, hospital admission, and overall survival) gives a comprehensive assessment of the impact of nutritional vulnerability across oncological care.

Limitations are acknowledged. The present study had a single‐centre design, a modest sample size, and an observational methodology, which precludes causal inference. The sample size in the high nutritional risk category was modest, partly due to a high proportion of breast cancer patients, who are at lower risk of malnutrition [41]. Muscle mass and body composition were not directly measured, preventing identification of sarcopenia or sarcopenic obesity [35, 46]. Toxicity outcomes were aggregated across symptom categories to create composite endpoints, and variability in grading may introduce measurement heterogeneity [47]. Participation was voluntary, with no restrictions on cancer type or treatments, limiting the ability to control for co‐founders. Although nutritional risk was associated with dose reduction, toxicity and mortality, malnutrition may also reflect disease burden, systemic inflammation, tumour biology [48], or treatment modality. Given the heterogeneity of the cohort, it was not possible to include all possible confounding variables, and as such, further research in a larger cohort is required to confirm the observations. Although there were associations between nutritional risk and adverse clinical outcomes, this study cannot determine whether the implementation of nutritional interventions would modify these outcomes [49]. The study aimed to identify gaps in dietetic service provision that warrant further evaluation and to develop a business case for a dedicated dietetic service for those attending for SACT.

### 4.2 Clinical Implications

Randomised controlled trials in oncology frequently face recruitment barriers, leaving many cancer subgroups without adequately powered evidence [50]. As a result, clinical decision‐making frequently depends on limited data, patient preferences, and local practice [51, 52]. Real‐world data are increasingly recognised as an important complement to RCTs, as they include heterogeneous populations with diverse prognoses and treatment trajectories [53]. Such data improve understanding of everyday clinical care and inform the design of future trials [51, 52].

The findings of the present study have important ramifications for routine oncological care. These can inform local policy decisions regarding the implementation of routine screening for those attending SACT and the need for multidisciplinary teams, including dietetics [29]. From our subsequent evaluation in this cohort, the PG‐SGA SF, compared to MUST, offered a pragmatic balance for sensitivity and specificity with the inclusion of patient‐reported outcomes and functional status [18]. By incorporating this screening tool, it could enable timely referral to dietetic services whilst also recognising other symptoms that require multidisciplinary input, such as functional decline, pain management, and psychological distress. Implementing nutritional screening at diagnosis could identify those at nutritional risk and guide appropriate interventions [33]. As nutritional status is potentially modifiable, integrating nutritional screening and intervention into oncology pathways may help to reduce treatment complications and improve survival outcomes [12]. Those identified as at nutritional risk would initiate referral to the appropriate healthcare professional, including dietitians working within multidisciplinary teams. The implications for healthcare services include policies and nutritional care pathways for nutritional screening in the outpatient setting. This is likely to have implications for the dietetic workforce, requiring more dietitians to be employed in oncology and palliative care. Future research should assess nutritional risk and access to specialised dietetic care in multinational, multicentred, RCTs to determine whether the implementation of nutritional screening and dietetic service provision differ internationally. Also, to evaluate whether implementing routine screening, referral pathways, and earlier dietetic intervention improves clinical outcomes, survival, healthcare utilisation, and cost‐efficiencies.

## 5 Conclusion

Nutritional risk is common among oncology day therapy patients and is independently associated with treatment‐related complications and overall survival. PG‐SGA SF provides prognostic value beyond traditional oncological predictors and supports routine nutritional screening in outpatient settings. Nutritional screening using the PG‐SGA SF identified those at nutritional risk, who experienced poorer clinical outcomes but were not under dietetic care.

The present study shows the prevalence of nutritional risk among oncology patients attending SACT, with 45.0% identified as at risk ≥ 4. Despite this, only 5.3% of the sample received dietetic input, emphasising a significant gap in meeting the nutritional needs of this cancer population. These findings also highlight the need for individualised nutritional care to support symptom management. Dietitians to be integrated into multi‐professional oncology and palliative care teams to help to optimise patient care and nutritional status, thereby improving treatment outcomes and QoL.

## Author Contributions

**Amy McLuskie:** conceptualisation, investigation, methodology, resources, formal analysis, investigation, data curation, validation, visualisation, writing – original draft, writing – review and editing. **Karen Angel:** conceptualisation, investigation, methodology, resources, validation, writing – review and editing. **Matthew Maddocks:** writing – review and editing. **Anne Finucane:** writing – review and editing. **Marie Fallon:** writing – review and editing. **Barry J. A. Laird:** conceptualisation, methodology, validation, investigation, writing – review and editing, supervision. **Michael S. Yule:** validation, formal analysis, investigation, data curation, writing – review and editing, visualisation, supervision.

## Conflicts of Interest

B.J.A.L. has received personal fees for consulting from Artelo, Actimed, Faraday, Kyona Kirin, Toray Pfizer, Abbott, and Nutricia. M.S.Y. has received an honorarium from MedAll for creation of educational resources.

## Data Availability

The data that support the findings of this study are available from the corresponding author upon reasonable request.
